# Arthroscopic and open reconstruction of the posterolateral corner of the knee have equally good clinical results: first results of a prospective 12-month follow-up study

**DOI:** 10.1007/s00402-024-05355-w

**Published:** 2024-05-25

**Authors:** H Fahlbusch, S Weiß, J Landenberger, F von Rehlingen Prinz, T Dust, R Akoto, M Krause, Karl-Heinz Frosch

**Affiliations:** 1 Department of Trauma and Orthopaedic Surgery, University Medical Center Hamburg-Eppendorf, Hamburg, Germany; 2 Department of Trauma Surgery, Orthopaedics and Sports Traumatology, BG Klinikum Hamburg, Hamburg, Germany; 3 Department of Orthopaedic Surgery, Trauma Surgery, and Sports Medicine Cologne Merheim Medical Center, Witten/Herdecke University, Ostmerheimer Str. 200, 51109 Cologne, Germany

**Keywords:** Posterolateral corner, Arthroscopy, Open surgery, PLC, Reconstruction, Knee, Posterior cruciate ligament

## Abstract

**Purpose:**

Arthroscopic reconstruction techniques for higher-grade posterolateral corner (PLC) injuries (Fanelli Type B, PoLIS LI-B) have not yet been validated in clinical studies. The open reconstruction technique described by Arciero is well-established and showed good restoration of joint stability in previous studies. This study aimed to compare clinical outcomes of this established open surgery technique to a newly developed arthroscopic technique in a prospective randomized clinical trial.

**Methods:**

Between 2019 and 2021, this study focused on chronic high-grade PLC injuries (Fanelli Type B, PoLIS LI-B). Group A consisted of patients treated with conventional open surgery following Arciero’s technique, while Group B included patients treated with Arciero’s arthroscopic technique. All cases underwent additional PCL reconstruction. After a minimum 12-month follow-up, clinical scores and objective stability assessments were compared between the groups.

**Results:**

In total, 26 (group A 12, group B 14) eligible patients with a mean follow-up of 14.9 ± 7.2 months were evaluated in the present study. Knee stability and patient-reported outcome scores (PROMS) were significantly improved when comparing pre- and post-operative values (*p* < 0.0001). No clinically relevant differences in PROMS (Lysholm: A 83.9 ± 11.4 vs. B 85.3 ± 13.8; IKDC: A 76.91 ± 12.6 vs. B 76.8 ± 15.7) were shown in both groups. Additionally, no statistically significant differences were detected between groups with respect to external rotation, range of motion and instrumental stability testing. Arthroscopic reconstruction showed significantly shorter operation time (*p* = 0.0109). There were no clinical failures or neurovascular complications of the surgical procedures.

**Conclusion:**

Both surgical techniques for isolated chronic PLC Fanelli Type B injuries significantly improved the knee stability, were equivalent with respect to PROMs and led to good clinical results. However, arthroscopic PLC reconstruction was associated with a shorter surgery time compared to open PLC reconstruction. Therefore, arthroscopic PLC reconstruction may be a viable option in the hands of an experienced surgeon.

**Level of evidence:**

Prospective cohort study, II.

**Supplementary Information:**

The online version contains supplementary material available at 10.1007/s00402-024-05355-w.

## Introduction

The posterolateral corner (PLC) consists primarily of the lateral collateral ligament (LCL) and the popliteus complex (PTC), which includes the popliteus muscle tendon (PLT) unit and the arcuate complex. The arcuate complex is formed by the popliteofibular ligament (PFL), the fabellofibular ligament and the popliteomeniscal fibers. Functionally, the popliteus complex serves as the most important static and dynamic stabilizer against external tibial rotation and posterior translation [1–3], whereas the LCL primarily provides stability against varus forces [4]. For posterolateral rotational instability (PLRI), Fanelli et al. [5] described a type B injury pattern marked by increased external rotation (ER), slight varus relaxation, and excessive posterior laxity, indicative of damage to PLT, PFL, LCL, and PCL. Weiler et al. [6], in a subsequent development, introduced the Posterolateral Instability Score (PoLIS) aligned with the aforementioned injury pattern, specifically PoLIS LI-B, presenting an innovative framework for evaluating injury severity and surgical decision-making.

Many arthroscopic reconstruction techniques have been proposed for restoring stability while utilizing the benefits of arthroscopic compared to open surgeries [7]. An arthroscopic approach can offer a better visualization of anatomic landmarks, avoid surgical morbidity and iatrogenic neurovascular injury, and provide the possibility of simultaneous arthroscopic PLC and PCL reconstruction [8–10]. In a biomechanical study arthroscopic anatomic reconstruction of the PLC restored nearly normal stability of the knee [11]. Novel arthroscopic techniques for anatomical reconstruction in higher-grade instabilities (Fanelli Type B) based on Arciero’s and LaPrade’s procedures, have been described by Frings et al., and Kolb et al. [12, 13]. Recently, both arthroscopic procedures were clinically compared for the treatment of type B injuries with promising results, showing sufficient restoration of posterolateral rotational instability, varus instability and posterior drawer through both techniques [14].

To our knowledge, no study has compared the clinical outcomes between arthroscopic and open PLC reconstruction in patients with Fanelli type B injuries. The aim of this study was to compare the arthroscopic anatomical PLC reconstruction technique described by Frings et al. [12] with the open conventional technique described by Arciero [15]. We hypothesized that both procedures could equally provide sufficient restoration of posterior, lateral, and external rotational stability and a comparable clinical outcome.

## Materials and methods

### Patient population

The study design was approved by the local ethics committee and an informed consent was obtained by each patient (2021-100677-BO-ff). All patients were informed about the available treatment options and provided their preoperative agreement to undergo the procedure explained to them.

A prospective study included 26 patients with high-grade posterolateral corner injuries (Fanelli Type B, PoLIS LI-B [6]) between 2019 and 2021. These injuries were treated either arthroscopically with reconstruction according to Frings et al. [12] (Group A) or open with Arciero’s conventional technique [15] (Group B).

**Fig. 1:**
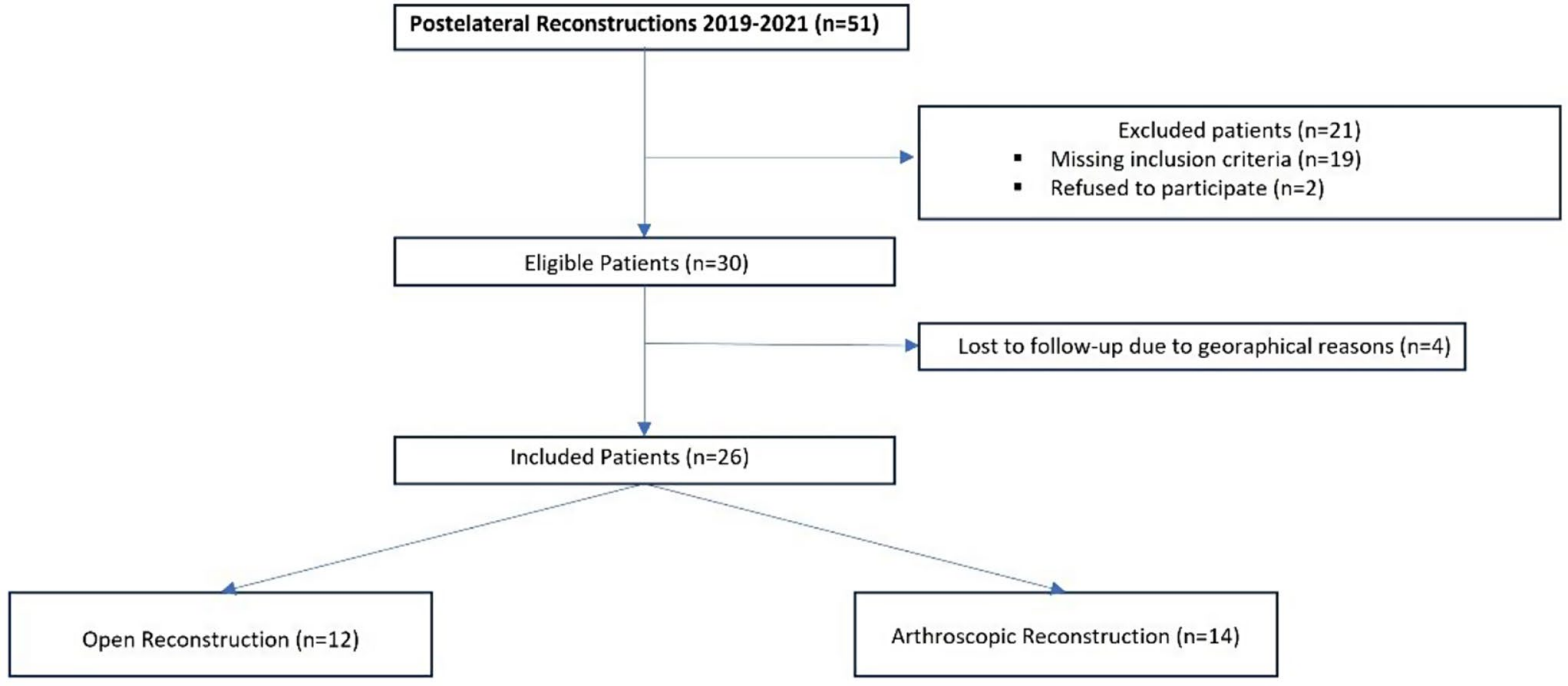
Flow chart for patients included in this study

Only patients presenting with chronic injuries (> 6 weeks), a combination of varus and posterolateral instability and additional posterior instability due to injury to the posterior cruciate ligament were included (see Fig. 1). The primary diagnosis was made by imaging (MRI, stress radiographs) and physical examination to assess ligamentous instability. Exclusion criteria were patient age under 18, obese patients (grade II according to WHO definition with BMI > 35 km/m^2^), coronal and sagittal malalignment, peroneal nerve injuries, higher- or lower-grade posterolateral corner injuries, additional ligamentous injury (Anterior cruciate ligament, Medial ligament complex) and additional affected structures (e.g. biceps femoris muscle, tendon rupture, iliotibial band injury and fractures at lower extremity).

### Preoperative clinical testing

The preoperative examination included ROM, Varus stress test, the posterior drawer test, and assessments of internal and ER at 30° and 90° of knee flexion (Dial test). The Dial test was considered positive at 30° if there was ≥ 10° more ER on the injured side, and positive at 90° if this side-to-side difference remained or increased. Additionally, clinical examination was conducted to assess lateral gapping by applying varus stress at full extension and 20°–30° of flexion. The clinical degree of varus instability was qualitatively compared with the contralateral side using the Hughston classification (negative, grade I - mildly positive, grade II - positive, grade III - severely positive) [16]. Only patients with a positive dial test at both knee flexion angles and mild varus gapping (mildly positive) were included (Fanelli B). Stability testing was conducted in pre-examination and during the anaesthetic examination.

### Surgical management

**Fig. 2:**
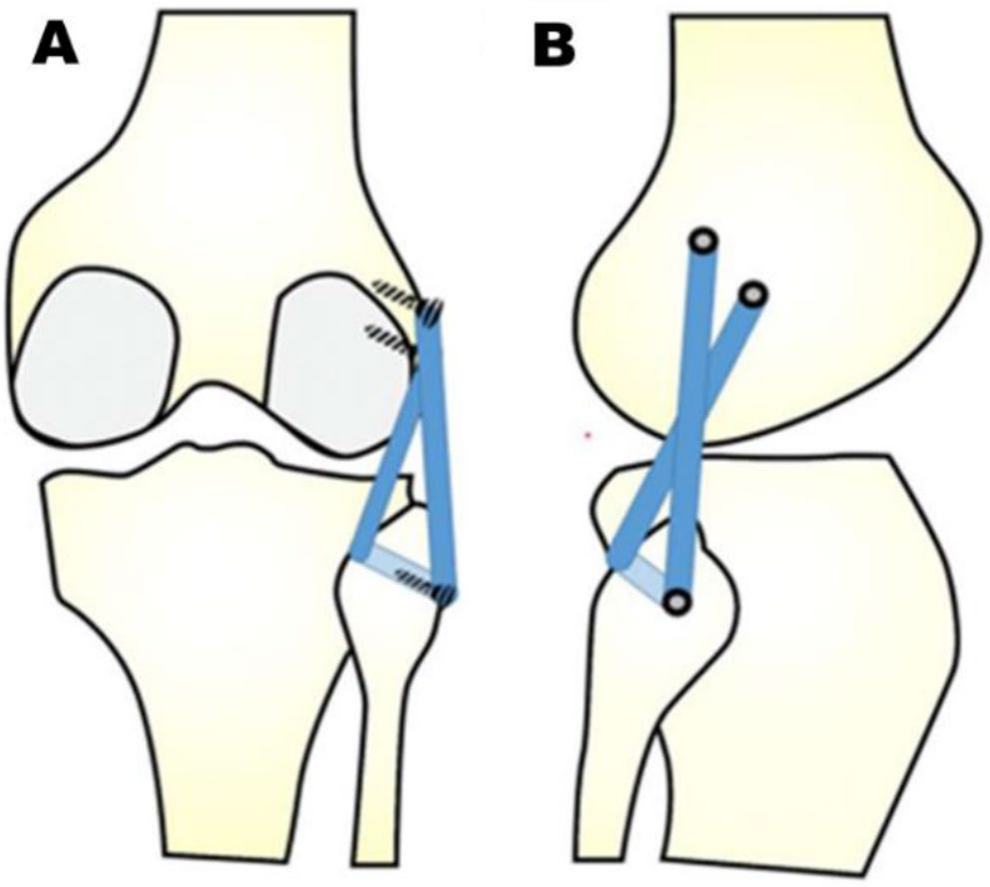
Schematic drawing of Arciero’s fibula-based, single-graft reconstruction technique for posterolateral corner reconstruction in posterior (**A**) and lateral view (**B**) modified from Weiss et al. [14]

**Fig. 3:**
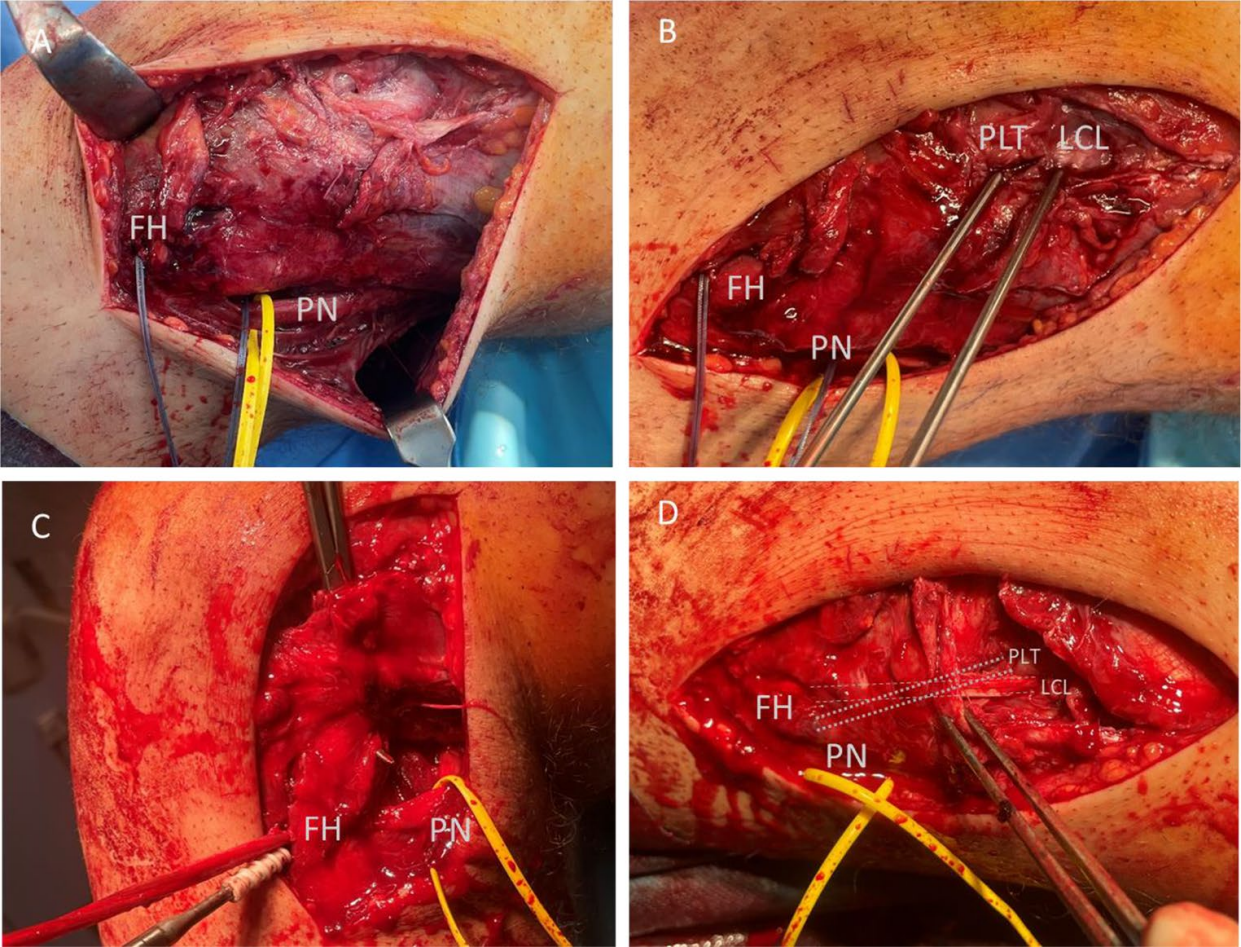
Intraoperative situs showing Arciero’s open technique for a 23-year-old patient who presented 7 weeks after a bike accident with a torn PCL and femoral avulsion of PLT, resulting in posterolateral rotational instability (Fanelli B). (**A**) The fibula head (FH) was shuttled with a suture. The peroneal nerve (PN) was exposed and marked with a vessel loop. (**B**) Two guidewires are introduced into the femoral native origins of the popliteus tendon (PLT) and lateral collateral ligament (LCL). (**C**) An autologous gracilis tendon graft is shuttled into the fibula head drilling canal and fixated with an interference screw. (**D**) The graft after fixation in the LCL and PLT tunnel

The type of surgical procedure was determined by block randomization. Initially, a brief arthroscopy was performed to rule out meniscal tears and any associated chondral lesions. Meniscal repair was possible in all cases. The arthroscopic anatomical reconstruction procedure introduced by Frings et al. [12] (Group A) and Arciero’s open technique [15] (Group B) were described in detail before. Arciero’s technique is a fibula-based technique with an anatomic transfibular tunnel placement, from anterolateral to posteromedial, in accordance with the native functional anatomy of the LCL and PLT (Figs. 2 and 3).

In addition, PCL reconstruction was arthroscopically performed during the same procedure. Hamstring tendon autografts were used in all cases. The duration of surgery was evaluated (starting from skin incision, including tendon harvest and all arthroscopic procedures until skin closure).

### Rehabilitation

Peripheral nerve block anesthesia was used in all cases. Physical therapy adhering to standardized protocols was initiated 48 h after the operation. For a period of 12 weeks, stabilizing braces with posterior tibial support (Jack PCL Brace; Albrecht, Bernau am Chiemsee, Germany) were worn while limiting the range of motion for 6 weeks. For the initial 2 weeks following surgery, the knee was flexed to a maximum angle of 20°. Physical therapists performed passive knee flexion up to 45° in a prone position after drain removal. During weeks 3 and 4, patients received passive mobilisation up to 60° of flexion and up to 90° during weeks 5 and 6. The patients had a weight-bearing limit of 20 kg for six weeks. After six weeks, the patients were allowed to have a full range of motion and gradually started full weight-bearing.

### Postoperative clinical testing

Follow-up examination was conducted at least 12 month following surgery and included functional outcome scoring systems by Lysholm and International Knee Documentation Committee (IKDC). Subjective pain during exercise was quantified by visual analogous scale (VAS). Posterior tibial translation (PTT) measurement was conducted by Rolimeter-Test (Aircast, Neubeuern, Germany) to measure the posterior translation of the tibia in the technique according to Höher et al. [17]. Clinical examination of ROM and ER in 30° and 90° of flexion was performed with the use of a HALO Goniometer® (HALO, model HG1, HALO Medical Devices, Australia) [18]. Varus stability was tested clinically at 0 and 30 degrees to evaluate collateral ligament stability.

We defined postoperative clinical failure as non-traumatic re-rupture of the posterior cruciate ligament that was confirmed through magnetic resonance imaging (MRI) or arthroscopy, or through a side-to-side difference (SSD) greater than 6 mm in posterior drawer test or a dial test SSD of ER greater than 10°.

### Statistical analysis

Statistical analysis was performed using GraphPad Prism 8 (San Diego, CA, USA). Data are presented as means and standard deviations (SD). Differences between the groups were calculated with the Student’s t test and Mann Whitney U-Test for non-parametric parameters. Difference within groups were calculated with paired Student’s t test. Categorical parameters were compared using Fisher’s exact text. A p-value < 0.05 was considered significant. An a priori sample size calculation indicated *n* = 24 patients (12 in each group) to detect a difference of 10 points in the clinical Lysholm score using G-Power (version 3.1.9.7., Heinrich Heine University, Düsseldorf) with a 𝛼 error of 5% and a power of 0.8.

## Results

### Patient demographics

Demographic data and surgical details of the included cases are displayed in Table 1. After an average follow-up of 14.9 ± 7.2 months, 26 patients were ultimately included in the study, 4 patients were lost to follow-up for geographical reasons. Duration of surgery was significantly longer in Group A patients than in group B (139.8 ± 31.8 vs. 183.2 ± 45.8 min; *p* = 0.0109).

**Table 1:** Demographic data

Characteristics	Total (*n* = 26)	Group A (*n* = 12)	Group B (*n* = 14)	*p*-value‡
Female Sex†	6 (23.1)	2 (16.7)	4 (28.6)	n.s.
Age in years^§^	35.2 ± 12.3	35.3 ± 11.2	35.2 ± 14.0	n.s.
Left Knee†	17 (65.4)	10 (83.3)	7 (50.0)	n.s.
BMI > 30 kg/m^2^†	5 (19.2)	2 (16.7)	3 (21.4)	n.s.
Time to surgery, in days^§^	877 ± 1579	1247 ± 2127	508 ± 625	n.s.
Follow-up, in months^§^	14.9 ± 7.2	13.7 ± 3.2	15.6 ± 10.0	n.s.
Duration of surgery, in min^§^	160.6 ± 44.3	139.8 ± 31.8	183.2 ± 45.8	**0.0109**
Hospitalization, in days^§^	5.3 ± 1.3	5.2 ± 1.4	5.5 ± 1.3	n.s.
Concomitant injuries†				n.s.
• Meniscal lesions	2 (8.0)	0	2 (53.8)	
• Cartilage damage	2 (8.0)	2 (16.7)	0	

*n* = 26; § mean ± SD; † n (in %); ‡ Shapiro–Wilk normality test and Kolmogorov–Smirnov test were performed to determine if the data were normally distributed, To compare both groups Student`s t-Test or Mann-Whitney U-Test were performed, Fisher’s exact test was used for comparison of binominal data; bold p value indicates statistical significance; SD standard deviation

### Patient reported functional outcome

Functional outcome scores at follow-up are shown in Table 2. There was no difference between the two groups according to VAS, Lysholm and IKDC scores. All participants were content with the overall outcome and would undergo the surgery again in hindsight. Among those who received arthroscopic treatment, six Patients (42.9%) were satisfied and eight Patients (57.1%) expressed high satisfaction. In the open group, two Patients (16.7%) expressed conditional contentment, whereas six Patients (41.7%) were satisfied, and six Patients (41.7%) were highly satisfied.

**Table 2:** Functional outcome scores at the follow-up

Parameters	Total (*n* = 26)	Group A (*n* = 12)	Group B (*n* = 14)	*p*-value‡
VAS (exercise)^§^				n.s.
• postoperative	1.9 ± 1.9	1.5 ± 0.9	2.3 ± 2.5	
Lysholm Score^§^				n.s.
• preoperative	60.2 ± 15.0	59.8 ± 18.9	60.5 ± 11.7	
• postoperative	84.6 ± 12.4	83.9 ± 11.4	85.3 ± 13.8	
Subjective IKDC score^§^				n.s.
• preoperative	51.9 ± 16.1	46.8 ± 19.4	60.5 ± 11.7	
• postoperative	74.6 ± 17.4	76.91 ± 12.6	76.8 ± 15.7	

*n* = 26; § mean ± SD; ); ‡ Shapiro–Wilk normality test and Kolmogorov–Smirnov test were performed to determine if the data were normally distributed, To compare both groups Student`s t-Test or Mann-Whitney U-Test were performed, p value indicates statistical significance; SD standard deviation; SD standard deviation; VAS visual analogous scale; IKDC International Knee Documentation Committee (IKDC)

### Clinical testing and instrumental stability testing

Regarding PTT, all patients except one had a preoperative SSD greater than 11 mm. In total, eleven patients had a preoperative SSD greater than 15 mm, with five patients in group A and six patients in group B. At the postoperative follow-up, there was no significant difference in SSD between the groups in terms of objective assessments of posterior drawer test, flexion or dial test results in ER at 30 or 90° flexion. Patients in both groups were postoperatively sufficiently clinically stable under varus loading (Varus stress-test: Negative 88.5%, mildly positive 11.5%) and showed a negative Dial test in 30° and 90° Flexion. The SSD of PTT, varus stability, SSD of ER and PROMS (Lysholm and IKDC-Score) were significantly improved in both groups when comparing preoperative and postoperative values (Groups A and B *p* < 0.0001). (see Table 3)

**Table 3:** Clinical examination and instrumental stability assessment pre- and postoperatively

Parameters	Total (*n* = 26)	Group A (*n* = 12)	Group B (*n* = 14)	*p*-value‡
Maximum Flexion^§^, in °				n.s.
• Preoperative	130.7 ± 14.4	129.3 ± 15.8	132.0 ± 13.3	
• Postoperative	129.1 ± 6.7	131.0 ± 5.1	127.1 ± 7.8	
Flexion SSD^§^, in °				n.s.
• postoperative	8.2 ± 4.9	8.5 ± 4.2	7.9 ± 5.8	
Dial test ER SSD^§^ at 30° Flexion, in °				n.s.
• postoperative	4.1 ± 4.9	4.4 ± 3.1	3.3 ± 6.5	
Dial test ER SSD^§^ at 90° Flexion, in °				n.s.
• postoperative	2.3 ± 3.7	2.8 ± 3.8	1.8 ± 3.7	
Varus Stress at 30° Flexion†				n.s.
• Preoperative				
• Grade II	26 (100)	12 (100)	14 (100)	
;• Postoperative				
• Negative	23 (88.5)	11 (91.7)	12 (85.7)	
• Grade I	3 (11.5)	1 (8.3)	2 (14.3)	
• Grade II	-	-	-	
• Grade III	-	-	-	
Posterior Drawer SSD^§^, in mm				n.s.
• preoperative	15.0 ± 2.4	15.1 ± 2.4	14.9 ± 2.4	
• postoperative	2.3 ± 1.2	2.6 ± 1.0	2.0 ± 1.4	
• Difference of Posterior Drawer (pre- and postoperative)	13.0 ± 2.7	13.1 ± 2.5	13.0 ± 3.1	

*n* = 26; § mean ± SD; † n (in %); ‡ Shapiro–Wilk normality test and Kolmogorov–Smirnov test were performed to determine if the data were normally distributed, To compare both groups Student`s t-Test or Mann-Whitney U-Test were performed, Fisher’s exact test was used for comparison of binominal data; SD standard deviation; ER External Rotation; SSD Side-to-side difference

### Complications

No patient experienced complications such as vascular or nerve injury, compartment syndrome, deep vein thrombosis or infection were reported. One patient in Group A reported joint stiffness (ROM 0-5-110°), which was resolved by arthroscopic arthrolysis after 46 weeks (ROM at last follow-up 0-0-125°). There were no cases of clinical failure in both groups.

## Discussion

The main finding in this prospective examination of chronic Fanelli B/PoLIS LI-B injuries was that the arthroscopic as well as the open technique showed sufficient restoration of posterior, rotational and lateral joint stability in clinical examinations and led to good (Lysholm-Score) and satisfying (IKDC-Score) clinical results.

Open procedures based on Arciero’s technique have shown significantly improved objective and subjective stability in higher grade instabilities, with equivalent clinical outcomes [19]. The arthroscopic equivalents for open Arciero reconstructions, as described by Frings et al. and Liu et al., respectively, provide viable options for those seeking arthroscopic procedures [11, 12, 14]. Arthroscopic PLC reconstruction requires profound knowledge of anatomic relations. However, it offers the benefit of visualizing all key structures, including the posteromedial aspect of the fibular head, for precise fibular drill tunnel placement [8, 20]. Some concerns have been raised regarding arthroscopic PLC reconstructions, such as the risk of tunnel misplacement and neurovascular injuries [21, 22]. However, Weiss et al. and our study’s clinical results did not show any neurovascular or tunnel-related complications [14]. Previous studies have demonstrated that the development of a transseptal portal and posterolateral arthroscopy can be safely performed with intraoperative knee flexion of 90° [8, 23–25]. The utilization of dependable soft-tissue and osseous reference points located at the fibular head allows for accurate arthroscopic anatomic fibular tunnel placement in the context of PLC surgery, consequently presenting a reduced probability of tunnel misalignment when contrasted with conventional open surgical methodologies [26]. Additionally, arthroscopic procedures circumvent the necessity for common peroneal nerve preparation and visualization, reducing the risk of nerve injury and saving time [27]. Notably, no instances of peroneal nerve damage were observed in either group in this study, indicating that both procedures are equally safe. We observed a shorter operative time in the arthroscopic group. A possible explanation for this difference could be the better arthroscopic visualisation of key structures and anatomical landmarks, leading to faster tunnel placement, apart from improved wound closure time.

To the best of our knowledge this is the first study to make a direct comparison between arthroscopic and open techniques regarding Fanelli B/PoLIS LI-B injuries. Overall our results align with previous studies that compared PROMS and restoration of stability, and lead to positive postoperative outcomes [14, 28–33]. Li et al. [30] conducted a study on PLC injuries, including 27 type A, 10 type B, and 12 type C patients, with 21 acute cases and 28 chronic cases. They used different reconstruction and repair techniques of the PLC and PCL single bundle reconstruction and followed up after a mean of 31.5 ± 9.3 months. The study showed that the PTT of SSD improved from 18.4 ± 9.2 mm to 5.2 ± 5.0 mm, and ER of SSD decreased from 18.0° ± 14.4° to 1.2° ± 7.5°. The same study group compared arthroscopic and open reconstruction of the PLT in combination with PCL reconstruction and showed no difference in PROMS and stability between the two groups of patients (SSD of PTT 4 and 5 mm and ER of 1 and 3°) [31]. Recently, Helal et al. [32] reported on a case series with a mean follow-up of 25 months. They found that 11 patients who underwent a reconstruction of the PLC and PCL had comparable results in external rotation (SSD of ER decreased from 16.7° to 3.5° postoperatively) and clinical outcomes (Lysholm/ IKDC 81/78 points). According to their report, not all of their patients had their varus stability sufficiently restored, whereas in our patients, only three had mildly positive varus instability (11.5%). This aligns with the findings of Sanders et al. [34], who reported that 88.5% of patients had grade 0 varus laxity, while 9.8% had grade I laxity, and 1.7% had grade II laxity. Van Gennip et al. [29] also demonstrated residual varus laxity but good clinical outcomes (Lysholm/IKDC 86/79 points) in 5 Fanelli C and 6 Fanelli B patients who underwent PLC reconstruction with a Larson technique in addition to PCL reconstruction, with a 2-year follow-up. Variations in surgical techniques may account for the difference in Varus stability. It is possible that Arciero’s anatomical technique is more effective in restoring stability than other non-anatomical techniques [35]. However, it is important to note that our study did not include an objective varus stability assessment, and the inclusion of Fanelli C patients in previous studies limits direct comparison. Overall, our results demonstrate that the anatomical technique of Arciero effectively restores the PLC, resulting in comparable PROMS and SSD of ER and PTT when compared to non-anatomical surgical techniques.

Arthrofibrosis is a commonly observed complication in multiligament knee injuries [36]. In this study, the incidence of arthrofibrosis was at 4% (1/26), which is comparable to other PLC-reconstructing studies [31, 32].

Overall, our study adds to the growing body of evidence that supports the feasibility and efficacy of arthroscopic PLC reconstruction for Fanelli B/PoLIS LI-B injuries. Arthroscopic care offers potential benefits, which were demonstrated by our study in terms of slightly increased patient satisfaction and shorter time of operation. However, the arthroscopic technique has a flat and long learning curve. While both arthroscopic and open techniques demonstrated favourable clinical outcomes and stability restoration, the choice of approach should be based on the surgeon’s experience, patient characteristics, and specific injury characteristics. Further research is warranted to explore the learning curve and long-term outcomes associated with arthroscopic PLC reconstruction, as well as to compare different arthroscopic techniques and their outcomes.

### Limitations

Scientific conclusions based on this study are limited by the relatively small number of cases due to the rare incidence of these complex injuries. In addition, matched-pair analysis was not possible due to the rarity of PLC injuries. All arthroscopic procedures were carried out by a single surgeon, whereas open surgeries were conducted by multiple surgeons. Although the inclusion and exclusion criteria were precise, the time between injury and surgery varied, but was always > 6 weeks and therefore considered as a chronic injury. Clinical follow-up of anterior-posterior stability testing was performed with the Rolimeter instead of stress radiographs, and varus/valgus instability was detected by clinical examination rather than stress radiographs. Long-term follow-up is needed to validate the concept of arthroscopic PLC reconstruction, as recent studies have shown promising results.

## Conclusion

Both surgical techniques for isolated chronic PLC Fanelli Type B injuries significantly improved the knee stability, were equivalent with respect to PROMs and led to good clinical results. However, arthroscopic PLC reconstruction was associated with a shorter surgery time compared to open PLC reconstruction. Therefore, arthroscopic PLC reconstruction may be a viable option in the hands of an experienced surgeon.

### Electronic supplementary material

Below is the link to the electronic supplementary material.

Supplementary Material 1

Supplementary Material 2

Supplementary Material 3

Supplementary Material 4

Supplementary Material 5

Supplementary Material 6

Supplementary Material 7
