# Supplementary material for: Arthroscopic and open reconstruction of the posterolateral corner of the knee have equally good clinical results: first results of a prospective 12-month follow-up study
Source: Arch Orthop Trauma Surg. 2024 May 25;144(6):2745–52. doi: 10.1007/s00402-024-05355-w (PMC11211137; doi:10.1007/s00402-024-05355-w)
Supplement: Supplementary file 7 — Supplementary Material 7 [file 402_2024_5355_MOESM7_ESM.pdf]

Springer

Disclosure of potential conflicts of interest

Authors must disclose all relationships or interests that could have direct or potential influence or impart bias on the work. Although an author may not feel there is any conflict, disclosure of all relationships and interests provides a more complete and transparent process, leading to an accurate and objective assessment of the work. Awareness of a real or perceived conflicts of interest is a perspective to which the readers are entitled. This is not meant to imply that a financial relationship with an organization that sponsored the research or compensation received for consultancy work is inappropriate. For examples of potential conflicts of interests *that are directly or indirectly related to the research please* visit:
springer.com/gp/authors-editors/journal-author/journal-author-helpdesk/before-you-start

All authors of papers submitted to AOTS (name of journal) must complete this form and disclose any real or perceived conflict of interest.

Please complete one form per author. The corresponding author collects the conflict of interest disclosure forms from all authors. The corresponding author will include a summary statement in the text of the manuscript in a separate section before the reference list, that reflects what is recorded in the potential conflict of interest disclosure form(s). Please note that you cannot save the form once completed. Kindly print upon completion, sign, and scan to keep a copy for your files.

The corresponding author should be prepared to send potential conflict of interest disclosure form if requested during peer review or after publication on behalf of all authors (if applicable).

☑ I have no potential conflict of interest.

Category of disclosure	Description of Interest/Arrangement

Article title Clinical outcome of arthroscopic versus open posterolateral corner reconstruction of the knee

Manuscript No. (if you know it) __________

Author name Jakob Landenberger

Are you the corresponding author? ☐ Yes ■ No

Herewith I confirm that the information provided is accurate.

Author signature __________ Date 27th of September, 2023
